# A 31-year-old male with a plasmacytoid dendritic blast cell neoplasm

**DOI:** 10.3332/ecancer.2024.1806

**Published:** 2024-11-29

**Authors:** Danay Caballero Hernández, Darío Álvaro Rueda, Leticia Rapan, Marcelo Iastrebner, Miguel Sorrentino

**Affiliations:** 1 Bone Marrow Transplant Service, Sanatorio Sagrado Corazón, Buenos Aires, CP 1039, Argentina; 2 Internal Medicine Service, Sanatorio Sagrado Corazón, Buenos Aires, CP 1039, Argentina

**Keywords:** myeloid leukaemia/classification, allogeneic hematopoietic stem cell transplantation, immunophenotyping, dendritic cells/pathology

## Abstract

Plasmacytoid blast dendritic cell neoplasm is a rare subtype of acute leukaemia that represents less than 1% of haematologic neoplasms. It is characterised by skin involvement and leukaemic dissemination in the rest of the body. The immunophenotype is represented by the expression of CD4, CD56 and CD123. Due to its low incidence, there is no standardised treatment. For most authors, acute lymphoblastic leukaemia) regimens with or without consolidation with allogeneic transplantation seem to be the most appropriate. We present the case of a 31-year-old male with a history of von Willebrand’s disease, who was diagnosed with plasmacytoid blast dendritic cell neoplasm with central nervous system involvement. After receiving first-line immunopolychemotherapy with rituximab, the patient achieved complete haematologic remission with the high-dose ara-C regimen. Subsequently, he consolidated with allogeneic haploidentical transplantation.

## Introduction

Plasmacytoid blast dendritic cell dendritic cell neoplasms were first described by Kameota et al. in 1988. Twenty years later they were recognised by the WHO as a subtype of myeloid neoplasm [1,2].

Blastic plasmacytoid dendritic cell neoplasms (BPDCNs) are a group of rare and aggressive diseases, accounting for less than 1% of haematologic malignancies. They have a bimodal incidence with a peak in those younger than 20 years and another in those older than 60 years. Their evolution is aggressive, with violaceous nodular skin lesions, splenomegaly, bone marrow (BM) infiltration and frequent involvement of the central nervous system (CNS) [3–9].

There are two types of BPDCN, classical and immunoblastoid (35% of the total). Morphologically the cells usually show cytoplasmic pseudopodia, a finding ‘similar to a ping-pong paddle’ [10].

Recognition of the immunophenotype of the neoplastic cell is important for its diagnosis, which is characterised by the expression of CD4, CD56, CD123 and TCL1, with negative lineage-specific antigens such as CD34, MPO or CD3. HLA-DR, CD303 (BDCA2), BDCA4 and positive lineage-specific antigens such as CD2AP were found whilst CD11c was negative [2, 3, 5, 7].

We present the case of a male patient with a history of von Willebrand’s disease (VWD) who presented a plasmacytoid dendritic blast cell neoplasm, which was treated with immunopolychemotherapy and consolidated with allogeneic BM transplantation.

## Clinical case

**Figure 1.**
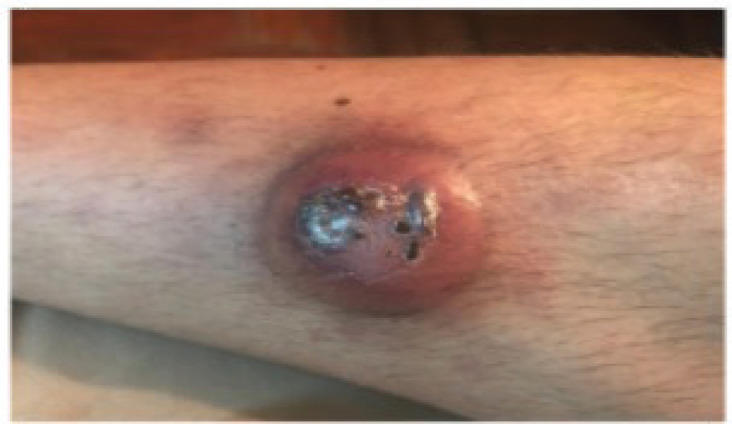
A nodular erythematous-violaceous lesion with central crusts is observed in the right pretibial region.

A 31-year-old male with a history of VWD type 2N was consulted for the presentation of a violaceous nodule in the right pretibial region of 1 month of evolution that increased in size with central necrosis and ulceration (Figure 1).

**Figure 2.**
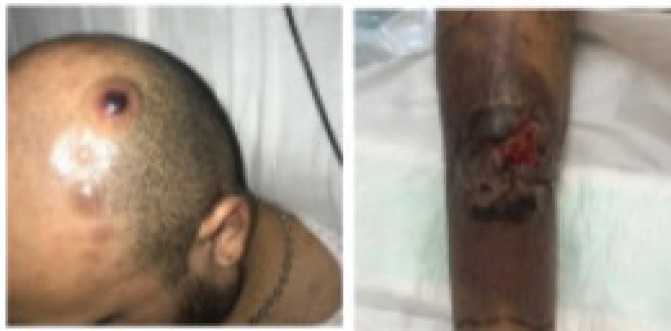
Multiple nodular lesions are observed on the scalp. The image on the right shows the evolution of the pretibial lesion, with increased erythema and necrotic tissue.

During his study, new lesions with similar characteristics to the tibial lesion appeared on the trunk and scalp, in addition to a right inguinal tumour with violaceous erythema extending towards the thigh (Figure 2).

On admission, the patient had white blood cells of 12,400/mm^3^ and platelets of 105,000/mm^3^. The CT scan revealed adenomegaly in the retroperitoneum, iliac chains and right inguinal region, the latter with a size of 60 × 30 mm. The leukocytosis rate increased, reaching a maximum value of 105,000/mm^3^ at 1 week of admission.

The peripheral blood smear showed 80% large immature cells, with a high nuclear-to-cytoplasm ratio, the presence of nucleoli, nuclear indentations, cytoplasm with vacuoles and irregular prolongations.

**Figure 3.**
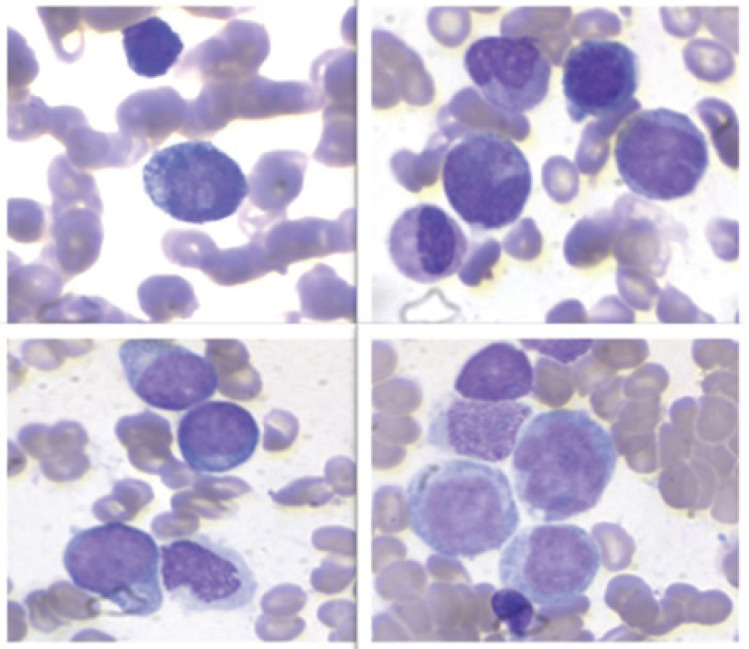
The bone marrow aspiration at 10x magnification showed monomorphic hypercellularity, with 90% infiltration by immature cells with intracitoplasmic pseudopodia, resembling a ping pong paddle.

The 10× BM aspirate showed monomorphous hypercellularity, with 90% infiltration by immature cells (Figure 3).

Flow cytometry (FC) identified two neoplastic populations with different phenotypic characteristics. The first population represented 24.6% of the cells, with positivity for CD20, CD19, CD38, CD10, CD45, HLA-DR, cytoplasmic lambda, CD81 and CD79a, and negativity for TdT and CD138. The second population represented 31.9%, and showed positivity for CD38, CD45, CD36, CD33, HLA-DR, CD117, CD7+ and CD71, and negativity for CD34, CD19, CD10, CD20, TdT, CD3c, CD3s, CD79a, MPO, CD138, CD41, CD61 and CD42b.

In the first population, CD19, CD10, CD20 and cytoplasmic lambda chain markers were indicative of a clonal lymphoid population. In the second population, CD117, HLA-DR and CD33 markers were indicative of an immature myeloid lineage.

A biopsy of the right inguinal node showed medium to large lymphoid proliferation with obvious nucleoli and scant cytoplasm. Immunohistochemistry was positive for CD123, CD68, CD33, CD56 and CD4, as well as CD43 and BCL2. It showed negativity for CD3, TdT, CD117, TIA-1 and granzyme. Lymphoid markers were positive in a scattered number of cells (PAX-5, CD20, CD10 and CD79+). The proliferation index of the MIB-1 marker was 80%.

The cytogenetic study by G-banding analysed 20 metaphases: 46XY, ins(6;8)(p21;q22q24), del(8)(q22),del(9)(q13)[2];46,XY,ins(6;8) (p21;q22q24), del(8)(q22). The two identified recurrent alterations were insertions on the short arm of chromosome 6 and deletion of the long arm of chromosome 8. FISH with IGH/c-MYC probe revealed a normal marker pattern.

Cerebrospinal fluid FC showed infiltrating cells with positivity for CD33, CD36, CD123 and CD71 and negative for CD34. Fundus examination and brain MRI with gadolinium were normal.

He was treated with rituximab, cyclophosphamide, vincristine, adriamycin and dexamethasone in combination with rituximab and high-dose methotrexate and ara-C (R-hyper-CVAD/R-MTX-araC schedule), and triple intrathecal therapy twice weekly. On day 21, evaluation of the patient had incomplete haematologic remission (CR) with 7% blasts by morphology and FC-positive measurable residual disease (MRD) of 0.18% cells with plasmacytoid dendritic cell phenotype, with no lymphoid clone present at diagnosis. Although the cerebrospinal fluid was negative.

CR was achieved after receiving the high-dose ara-C regimen. Subsequently, she consolidated with related haploidentical allogeneic transplantation. Conditioning was performed with cyclophosphamide 14.5 mg/kg on day minus 6 and minus 5, fludarabine 30 mg/m² from day minus 6 to minus 2 followed by total body radiotherapy at a dose of 2 Gy on day minus 1 post-transplant. On post-transplant day 3, he received cyclophosphamide 50 mg/kg, and started immunosuppression with mycophenolate and tacrolimus on day 5 (original Baltimore protocol). Currently, the patient is in complete remission with negative MRD and 100% chimerism for 7 years. He required FVIII/FVW administration with each invasive procedure. During the second month post-transplantation, he presented moderate acute graft-versus-host disease (GVHD) that responded to corticosteroids, and at 8 months, mild chronic GVHD with skin involvement, also successfully treated with corticosteroids. She discontinued immunosuppression 6 months after transplantation.

## Discussion

BPDCN is a rare form of acute leukaemia that usually presents with skin lesions and adenopathy. The immunophenotype is characterised by the expression of CD4, CD56, CD123 and TCL1, as in the present case [7, 9].

The clinical findings and the immunophenotyping study allowed us to reach the diagnosis of BPDCN [5]. Most patients show alterations in the karyotype, with complex karyotype in more than 75%, as in our case, where the insertion in the short arm of chromosome 6 and the deletion of the long arm of chromosome 8 were detected. Rearrangements involving 8p24 where the cMyc gene is located are evidenced in 38% of the cases. The alteration of the cMyc gene occurs more frequently in older adults, with a median age of 70 years, predominantly in males, and in 100% of the cases with involvement of BM [3, 11, 12].

Synchronous or metachronous myeloid neoplasms such as chronic myelomonocytic leukaemia, myelodysplastic syndrome or acute myeloid leukaemia (AML) have been described in BPDCN [5]. Due to the presence of a lymphoid clone in the FC, we considered the possibility of a synchronous lymphoid neoplasm, which motivated us to use rituximab in the initial scheme. Another differential diagnosis that was considered was bilinear mixed phenotype acute leukaemia, but the absence of expression of immaturity antigens (CD34, C117, HLA-DR,) distanced us from this possibility. We considered the diagnosis of CD56-positive AML with aberrant expression of lymphoid antigens, since the leukaemic cells expressed CD56+, heterogeneous CD33+ and weak CD117+, but CD4 and CD123 positivity, added to the clinical presentation of the patient, made it unlikely [13].

Regarding morphology, microvacuoles are frequently observed in BPDCN, probably due to glycogen deposits, and are usually located around the nucleus resembling a string of pearls; also Periodic acid–Schiff (PAS)-negative macrovacuoles have been described as an infrequent morphological finding. Pseudopodia are usually present, and they can be large or small [3, 10]. In our case, large pseudopodia were observed, and isolated. Isolated frequent macro vacuoles were present, and PAS staining could not be performed [3].

Skin lesions are the hallmark of the disease, present in more than 90% of cases, may precede BM involvement by up to 2–3 months, and rarely is involvement confined to the skin [3, 7]. Three typical presentations have been described: brown or purple nodules (73%), in the form of brown to purplish ‘bruise-like’ infiltrated patches (12%), or disseminated and mixed lesions [13]. Our patient initially showed violaceous nodules and with disease progression mixed lesions.

The presence of adenomegaly and BM involvement are frequent, unusually they present as acute leukaemia. CNS involvement is common at diagnosis and may be asymptomatic as in our patient [5, 9].

There is no standardised treatment for BPDCN given its low incidence. Polychemotherapy regimens of acute lymphoblastic leukaemia (ALL), AML or lymphomas have been used. For most authors, ALL regimens with or without consolidation with TALO appear to be the most appropriate. Although there are no randomised studies, retrospective series, such as that of Murthy *et al* [9] and Kharfan-Dabaja *et al* [8], among others, suggest consolidation with TALO in the first CR, when the patient is eligible and the donor is available [8, 9, 14–17].

In a cohort of patients published by Italian authors, they evaluated responses with ALL and AML-type regimens, observing better responses with the former [6].

The strategy of myeloablative schemes followed by TALO in the first CR in *fit* patients has a 3-year overall survival rate ranging from 52% to 74%. Although there is evidence of the use of autologous transplantation after achieving CR, this option offers an overall survival rate of 1 year at 11%, therefore, it is not recommended [8, 9, 16].

The R-hyper-CVAD/ R-MTX-araC scheme seemed to be the most appropriate, as it was useful for treating BPDCN and the other differential diagnoses raised, with the addition of Rituximab due to CD20 positivity. However, the best response was obtained with the AML-type regimen, even achieving MRD negativity. The conditioning regimens chosen by other authors include myeloablative regimens with cyclophosphamide in fit patients. Treatment of occult CNS involvement improves response to treatment and our, patient was negative for FC in cerebrospinal fluid with triple intrathecal therapy. The R-H-CVAD/R-MTX-araC scheme has been used by other authors in patients with BPDCN and (t6;8) (p21;p24) without achieving responses [12, 14].

At present, Tagraxofusp is recommended as remission induction therapy in BPDCN, it is a cytotoxin that binds directly to CD123, it is not available in our country so it was not taken into account in the choice of treatment [9, 16].

The BCL2 inhibitor venetoclax also appears to be a promising drug in patients with refractory relapse, according to some recent publications [4, 6].

## Conclusion

BPDCN is a rare and aggressive neoplasm with an unfavourable prognosis. In our case, treatment with an AML-type scheme and consolidation with TALO in the first CR phase proved to be an adequate strategy.

## Conflicts of interest

The authors declare that there is no conflicts of interest in the preparation of this manuscript.

## Funding

No funding was received for this study.

## Author contributions

All authors have contributed to the preparation of the manuscript.
